# Severe Falciparum and Vivax Malaria on the Thailand-Myanmar Border: A Review of 1503 Cases

**DOI:** 10.1093/cid/ciad262

**Published:** 2023-05-05

**Authors:** Cindy S Chu, Marie Stolbrink, Daniel Stolady, Makoto Saito, Candy Beau, Kan Choun, Tha Gay Wah, Ne Mu, Klay Htoo, Be Nu, Arunrot Keereevijit, Jacher Wiladpaingern, Verena Carrara, Aung Pyae Phyo, Khin Maung Lwin, Christine Luxemburger, Stephane Proux, Prakaykaew Charunwatthana, Rose McGready, Nicholas J White, François Nosten

**Affiliations:** Shoklo Malaria Research Unit, Mahidol–Oxford Tropical Medicine Research Unit, Faculty of Tropical Medicine, Mahidol University, Mae Sot, Thailand; Centre for Tropical Medicine and Global Health, Nuffield Department of Medicine, University of Oxford, Oxford, United Kingdom; Shoklo Malaria Research Unit, Mahidol–Oxford Tropical Medicine Research Unit, Faculty of Tropical Medicine, Mahidol University, Mae Sot, Thailand; Shoklo Malaria Research Unit, Mahidol–Oxford Tropical Medicine Research Unit, Faculty of Tropical Medicine, Mahidol University, Mae Sot, Thailand; Centre for Tropical Medicine and Global Health, Nuffield Department of Medicine, University of Oxford, Oxford, United Kingdom; Division of Infectious Diseases, Advanced Clinical Research Center, Institute of Medical Science, University of Tokyo, Tokyo, Japan; Shoklo Malaria Research Unit, Mahidol–Oxford Tropical Medicine Research Unit, Faculty of Tropical Medicine, Mahidol University, Mae Sot, Thailand; Shoklo Malaria Research Unit, Mahidol–Oxford Tropical Medicine Research Unit, Faculty of Tropical Medicine, Mahidol University, Mae Sot, Thailand; Shoklo Malaria Research Unit, Mahidol–Oxford Tropical Medicine Research Unit, Faculty of Tropical Medicine, Mahidol University, Mae Sot, Thailand; Shoklo Malaria Research Unit, Mahidol–Oxford Tropical Medicine Research Unit, Faculty of Tropical Medicine, Mahidol University, Mae Sot, Thailand; Shoklo Malaria Research Unit, Mahidol–Oxford Tropical Medicine Research Unit, Faculty of Tropical Medicine, Mahidol University, Mae Sot, Thailand; Shoklo Malaria Research Unit, Mahidol–Oxford Tropical Medicine Research Unit, Faculty of Tropical Medicine, Mahidol University, Mae Sot, Thailand; Shoklo Malaria Research Unit, Mahidol–Oxford Tropical Medicine Research Unit, Faculty of Tropical Medicine, Mahidol University, Mae Sot, Thailand; Shoklo Malaria Research Unit, Mahidol–Oxford Tropical Medicine Research Unit, Faculty of Tropical Medicine, Mahidol University, Mae Sot, Thailand; Shoklo Malaria Research Unit, Mahidol–Oxford Tropical Medicine Research Unit, Faculty of Tropical Medicine, Mahidol University, Mae Sot, Thailand; Centre for Tropical Medicine and Global Health, Nuffield Department of Medicine, University of Oxford, Oxford, United Kingdom; Faculty of Medicine, Institute of Global Health, University of Geneva, Geneva, Switzerland; Shoklo Malaria Research Unit, Mahidol–Oxford Tropical Medicine Research Unit, Faculty of Tropical Medicine, Mahidol University, Mae Sot, Thailand; Shoklo Malaria Research Unit, Mahidol–Oxford Tropical Medicine Research Unit, Faculty of Tropical Medicine, Mahidol University, Mae Sot, Thailand; Shoklo Malaria Research Unit, Mahidol–Oxford Tropical Medicine Research Unit, Faculty of Tropical Medicine, Mahidol University, Mae Sot, Thailand; Shoklo Malaria Research Unit, Mahidol–Oxford Tropical Medicine Research Unit, Faculty of Tropical Medicine, Mahidol University, Mae Sot, Thailand; Mahidol–Oxford Tropical Medicine Research Unit, Faculty of Tropical Medicine, Mahidol University, Bangkok, Thailand; Department of Clinical Tropical Medicine, Faculty of Tropical Medicine, Mahidol University, Bangkok, Thailand; Shoklo Malaria Research Unit, Mahidol–Oxford Tropical Medicine Research Unit, Faculty of Tropical Medicine, Mahidol University, Mae Sot, Thailand; Centre for Tropical Medicine and Global Health, Nuffield Department of Medicine, University of Oxford, Oxford, United Kingdom; Centre for Tropical Medicine and Global Health, Nuffield Department of Medicine, University of Oxford, Oxford, United Kingdom; Mahidol–Oxford Tropical Medicine Research Unit, Faculty of Tropical Medicine, Mahidol University, Bangkok, Thailand; Shoklo Malaria Research Unit, Mahidol–Oxford Tropical Medicine Research Unit, Faculty of Tropical Medicine, Mahidol University, Mae Sot, Thailand; Centre for Tropical Medicine and Global Health, Nuffield Department of Medicine, University of Oxford, Oxford, United Kingdom

**Keywords:** *Plasmodium vivax*, *Plasmodium falciparum*, severe malaria, epidemiology

## Abstract

**Background:**

The northwestern border of Thailand is an area of low seasonal malaria transmission. Until recent successful malaria elimination activities, malaria was a major cause of disease and death. Historically the incidences of symptomatic *Plasmodium falciparum* and *Plasmodium vivax* malaria were approximately similar.

**Methods:**

All malaria cases managed in the Shoklo Malaria Research Unit along the Thailand-Myanmar border between 2000 and 2016 were reviewed.

**Results:**

There were 80 841 consultations for symptomatic *P. vivax* and 94 467 for symptomatic *P. falciparum* malaria. Overall, 4844 (5.1%) patients with *P. falciparum* malaria were admitted to field hospitals, of whom 66 died, compared with 278 (0.34%) with *P. vivax* malaria, of whom 4 died (3 had diagnoses of sepsis, so the contribution of malaria to their fatal outcomes is uncertain). Applying the 2015 World Health Organization severe malaria criteria, 68 of 80 841 *P. vivax* admissions (0.08%) and 1482 of 94 467 *P. falciparum* admissions (1.6%) were classified as severe. Overall, patients with *P. falciparum* malaria were 15 (95% confidence interval, 13.2–16.8) times more likely than those with *P. vivax* malaria to require hospital admission, 19 (14.6–23.8) times more likely to develop severe malaria, and ≥14 (5.1–38.7) times more likely to die.

**Conclusions:**

In this area, both *P. falciparum* and *P. vivax* infections were important causes of hospitalization, but life-threatening *P. vivax* illness was rare.

## Teaser

Among 94 467 patients with *Plasmodium falciparum* and 80 841 with *Plasmodium vivax* malaria, patients with *P. falciparum* were 15 times more likely to require hospital admission, 19 times more likely to develop severe malaria, and 14 times more likely to die.

Early in the 20th century, *Plasmodium vivax* was known as “benign tertian malaria” to differentiate it from the commonly fatal “malignant tertian” malaria caused by *Plasmodium falciparum* [1]. In the 1920s, early in the era of malaria therapy for neurosyphilis [2, 3], the lethal potential of *P. falciparum* was soon evident, so artificial infection with the safer *P. vivax* malaria became the therapy of choice. Even so, malaria therapy showed that patients who were already severely debilitated by neurosyphilis could die as a result of any of the human malaria infections—even *Plasmodium malariae* [4]. It was also recognized that *P. vivax* generally caused fever at lower parasite densities than *P. falciparum* [5].

The early descriptions of malaria from military and malaria therapy experiences, which created the “textbook” descriptions, referred usually to previously unexposed (ie non-immune) adults [6, 7]. They are more relevant today to malaria in travelers and less so to malaria in endemic areas where the population is exposed repeatedly to malaria infections. In low-transmission areas, malaria occurs at all ages, whereas in higher-transmission settings, with the acquisition of disease controlling immunity, malaria illness is largely confined to younger children. It is estimated that >90% of the deaths from severe malaria in the world are in African children. Nearly all are attributable to *P. falciparum* [8]. In higher-transmission settings, where asymptomatic patent malaria parasitemia is very common, it is difficult to distinguish malaria as the cause of illness from other illnesses with coincidental malaria parasitemia [9–11]. Even in low-transmission areas, a significant proportion of the community has asymptomatic parasitemia [12]. The mortality rate directly attributable to malaria is consequently overestimated—often by a substantial amount [13].

In recent years, as the global burden of malaria and the geographic extent of malaria-endemic areas have decreased, the number of reports of severe vivax malaria has increased markedly. It is unclear whether this represents a genuine rise, increased recognition, a lower threshold for the diagnosis, incorrect attribution, or selective reporting. The prevalence of severe *P. vivax* infections has varied markedly both across and within geographic regions [14–20]. Clinical manifestations reported in severe *P. vivax* malaria are similar to those reported in severe *P. falciparum* malaria [14]. They include pulmonary edema, severe anemia, shock, hypoglycemia, hepatic or renal dysfunction, neurologic dysfunction (cerebral malaria or multiple convulsions), and severe thrombocytopenia [15]. Extremes of age, comorbid conditions, and chloroquine resistance have been associated with an increased risk of severe vivax malaria [17–20]. As in the earlier malaria therapy experience, already debilitated patients are at greatest risk [3, 4].

One meta-analysis estimated that 1.1% of persons with symptomatic *P. vivax* infections had severe malaria (11 658 of 10 590 970 total cases). The case fatality was reported as 5% in patients with ≥1 severe manifestation [14]. Very few reports have come from the countries of the Greater Mekong subregion, where severe *P. vivax* malaria is considered rare [16]. These marked geographic differences, the lack of detailed prospective clinical studies, and the difficulty in establishing causality leave substantial uncertainty over the true incidence, prevalence, and outcomes of severe vivax malaria. Our retrospective review of all patients admitted to clinics and hospitals at the Shoklo Malaria Research Unit (SMRU) on the northwestern border of Thailand over a 16-year period was performed to provide a comparative assessment of prevalence and severity of illness caused by these 2 main malaria species.

## METHODS

### Study Population

This observational study was conducted by SMRU, which has operated malaria clinics and inpatient facilities along the northwestern Thailand-Myanmar border since 1986. The epidemiology of malaria in this region of hill forest and low seasonal malaria transmission has been studied in detail and reported elsewhere [21, 22]. The patient population comprised migrant workers and displaced persons of all ages of Burman and Karen ethnicities. Until 2012, patient numbers in the transmission season were very high, with nearly 200 consultations each day at one health clinic (50% with confirmed malaria). Before 2010, malaria was diagnosed based on either a *P. falciparum* specific rapid diagnostic test or a malaria smear, whereby parasite counts were provided for *P. falciparum,* and *P. vivax* infection was noted but not quantitated. After 2010, *P. vivax* parasite densities were quantitated. All patients with >4% *P. falciparum* parasitemia (hyperparasitemia) were admitted. Otherwise, patients were hospitalized at the physician’s discretion. All had a malaria smear performed. Pulse oximetry was not available before 2009, and was not performed routinely until 2015.

Three days of oral chloroquine (25 mg base/kg) was given for *P. vivax* malaria, and 3 days of oral artemisinin combination therapy (eg, mefloquine-artesunate, artemether-lumefantrine, or dihydroartemisinin-piperaquine) was given for *P. falciparum* malaria. As second-line treatment in *P. falciparum* malaria, or for treatment failure, 7-days of quinine or artesunate combined with doxycycline or clindamycin was given [23]. Primaquine radical cure for *P. vivax* was not prescribed routinely during the study period. Patients with >4% parasitemia were given artesunate (oral or intravenous, depending on the patient’s clinical condition), and completed 7 days of treatment with artemisinin combination therapy [23]. Other medical management included anticonvulsants to treat seizures, blood transfusions, oral or intravenous antibiotics, and intravenous fluids. Positive pressure ventilation and renal replacement therapies were not available. Full blood cell counts and biochemical and microbiology investigations were not available routinely before 2010.

### Study Methods

All patients attending SMRU outpatient clinics had an electronic data entry. Anonymized data from October 2000 to December 2016 provided the total number of outpatient consultations with a malaria diagnosis. For the same period, records of patients admitted to the SMRU hospitals with *P. falciparum* or *P. vivax* malaria were obtained from the inpatient electronic database [24]. To account for hospitalizations for post-delivery complications unrelated to malaria, post-partum women were defined as those from 1 calendar day up to <6 weeks from the date of delivery. Dates, age, sex, weight, medical history, presenting symptoms and their duration, vital signs, clinical examination, results of diagnostic tests performed, treatment, and discharge diagnoses were extracted.

Severe malaria diagnoses were based on the current broad World Health Organization (WHO) classification [25] and also analyzed using the stricter research definition, which excludes prostration or convulsions as severe malaria criteria [26] (Supplementary Table 1). Severe anemia was defined as in the WHO classification: hemoglobin ≤5 g/dL or hematocrit ≤15% in children <12 years old (<7 g/dL and <20%, respectively, in adults). Chest radiography was not available on site. Pulmonary edema was diagnosed clinically if pulse oximetry was <92% on room air or—if oximetry was unavailable—if the respiratory rate was elevated for age *and* chest examination findings were abnormal. If the patient was visibly jaundiced, the serum total bilirubin was assumed to be >50 µmol/L (>3 mg/dL). If a discharge diagnosis of renal failure was recorded, the patient hospital record was reviewed to determine the basis for the diagnosis. Parasite density thresholds for *P. vivax* were not used in the severity definitions [26].

### Statistical Analysis

Comparisons were made using χ^2^ or Fisher exact or Student *t* or nonparametric K-sample tests, as appropriate. Multivariable generalized linear modeling was used to assess the effects of age, sex, pregnancy and postpartum status, malaria species, and the presence of concomitant disease on whether the WHO severe malaria criteria were met [25, 26] for *P. vivax* compared with *P. falciparum*. Statistical analysis was performed using Stata 15.1 software (StataCorp).

### Ethical Review

Ethical approval was given by the Ethics Committee at the Faculty of Tropical Medicine, Mahidol University (TMEC 17–049), the Oxford Tropical Research Ethics Committee (OXTREC 28-09), and the Tak Community Advisory Board (20170729/TCAB-11).

## RESULTS

**Figure 1.**
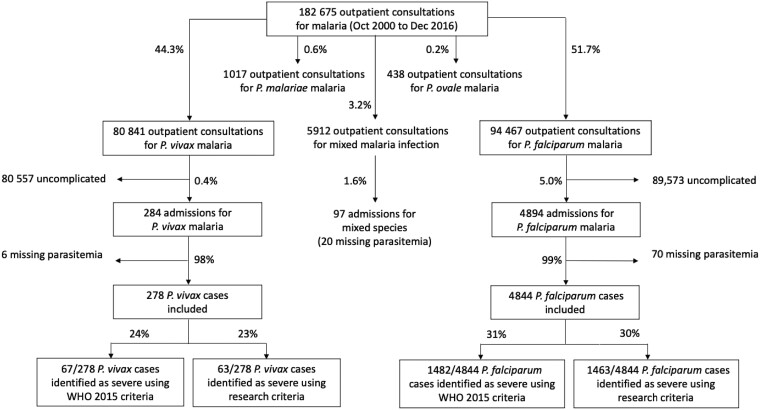
Flow diagram of malaria cases included. Excluded cases are not enclosed in an outlined box. Abbreviations: *P. falciparum, P. malariae, P. ovale,* and *P. vivax, Plasmodium falciparum, malariae, ovale,* and *vivax*; WHO, World Health Organization.

**Figure 2.**
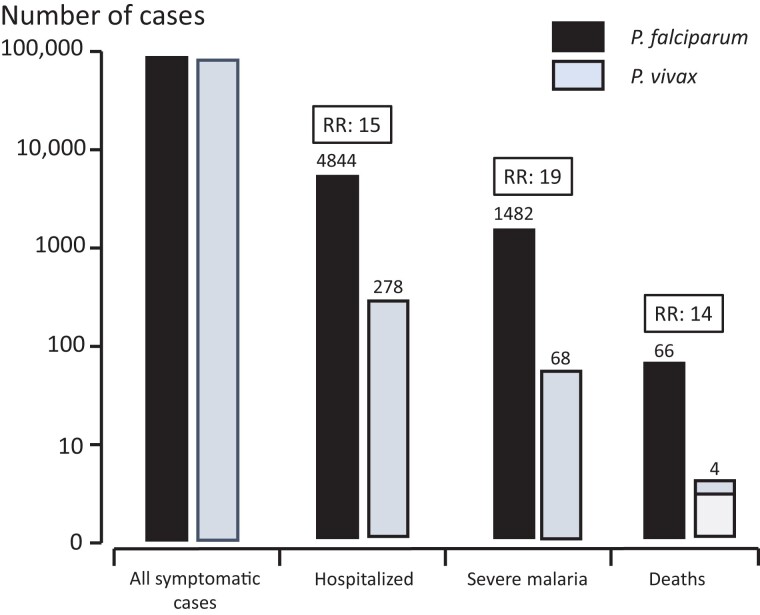
The relative risks (RRs) of hospitalization, severe malaria, and death for *Plasmodium falciparum* compared with *Plasmodium vivax.* In 3 of the 4 fatalities with *P. vivax* malaria (*white box*) the primary diagnosis was sepsis, and the causal role of malaria was uncertain.

Between October 2000 and December 2016, there were 80 841 consultations for *P. vivax* malaria, 94 467 for *P. falciparum*, 1017 for *P. malariae*, and 438 for *Plasmodium ovale.* The vast majority of patients (80 557 [99.6%] with *P*. *vivax* and 89 573 [95%] with *P*. *falciparum*) had uncomplicated illness. Mixed-species infections were documented in 5912 (3.2%) of the 182 675 symptomatic malaria infections. A total of 175 308 cases were included in this study; 278 patients (0.34%) were hospitalized with a diagnosis of severe *P. vivax* malaria, and 4844 (5.0%) were hospitalized for severe *P. falciparum* malaria (Figure 1). Thus, symptomatic *P. falciparum* infections were 15 (95% confidence interval [CI], 13.2–16.8) times more likely than *P. vivax* infections to require hospital admission (*P* < .001) (Figure 2). Parasite densities in patients hospitalized with *P. vivax* infections ranged from 16/µL to 189 028/µL (geometric mean, 2284/µL), and in those with *P. falciparum* infection they ranged from 16/µL to 2 409 007/µL (geometric mean,161 231/µL) (Table 1).

**Table 1.** Characteristics of Patients Admitted to Shoklo Malaria Research Unit Hospitals Between 2000 and 2016 With *Plasmodium vivax* or *Plasmodium falciparum* Malaria

Characteristic	Values by Patient Age Group, Median (Range)^a^						
	0–28 d	29 d to <1 y	1–5 y	6–10 y	11–15 y	>15 y	Total
*Plasmodium vivax*							
All records, no. (%)^b^	11 (4)	45 (16)	64 (23)	20 (7)	10 (4)	128 (46)	278 (100)
Male sex, no. (%)^c^	b5 (45)	24 (53)	33 (52)	11 (55)	6 (60)	26 (20)	105 (38)
Weight, kg	3.4 (1.5 to 4.4) [1]	6.5 (2.6–9.7)	10.0 (6.3–18) [1]	16.5 (11–28)	32.5 (25–49)	49 (30–71) [1]	26 (1.5–71) [3]
Temperature, °C	38.5 (36.5–39.8)	37.8 (36.3–40.0)	38.8 (36.5–40.7)	38.0 (36.0–40.3)	38.2 (37.4–40.1)	38.0 (35.9–41.2)	38.2 (35.9–41.2)
Pulse rate, beats/min	148 (90–180)	140 (90–188)	131 (98–199)	126 (84–160)	98 (72–120)	96 (66–148)	119 (66–199)
Respirations/min	48 (30–80)	46 (20–76)	39 (20–90)	32 (22–48)	26 (20–38)	28 (14–52)	32 (14–90)
Hematocrit, %	36 (29–47) [2]	27 (12–50) [2]	30 (9–44) [2]	29 (11–40) [4]	33 (12–43) [2]	33 (3–56) [8]	32 (3–56) [20]
Met severe anemia definition, no. (%)^c,d^	0	2 (4)	2 (3)	2 (10)	1 (10)^d^	11 (9)^d^	18 (6)
Parasitemia, geometric mean (range), parasites/µL	9871 (256–189 028)	1738 (16–125 600)	2719 (16–143 184)	1350 (48–58 027)	1713 (32–35 168)	2257 (16–105 504)	2284 (16–189 028)
*Plasmodium falciparum*							
All records, no. (%)^b^	2 (0.04)	88 (2)	1156 (24)	888 (18)	613 (13)	2097 (43)	4844 (100)
Male sex, no. (%)^c^	1 (50)	43 (49)	615 (53)	520 (59)	404 (66)	1447 (69)	3030 (63)
Weight, kg	2.5 (2.0–2.9)	7.0 (2.6–11)	12 (5.0–25) [1]	19 (8–50)	33 (16–58) [1]	50 (20–84) [6]	31 (2–84) [8]
Temperature, °C	36.9 (35.8–38.0)	37.8 (34.8–40.3)	38.1 (35.5–40.9)	38.2 (35.5–41.5)	38.1 (35.0–41.5)	38.0 (34.7–41.6) [3]	38.0 (34.7–41.6) [3]
Heart rate, beats/min	139 (120–158)	140 (100–190)	132 (60–200)	120 (64–200)	110 (64–160) [1]	100 (60–160) [35]	114 (60–200) [36]
Respirations/min	39 (34–44)	44 (26–90)	36 (14–80)	30 (14–60)	28 (18–52) [1]	26 (10–62) [35]	28 (10–90) [36]
Hematocrit, %	40 (26–54)	26 (9–45)	29 (6–61) [9]	33 (9–50) [5]	36 (8–54) [1]	37 (5–77) [23]	34 (5–77) [38]
Met severe anemia definition, no. (%)^c,d^	0	12 (14)	64 (6)	18 (2)	9 (1)^d^	64 (3)^d^	167 (3)
Parasitemia, geometric mean (range), parasites/µL	9655 (128–728 229)	124 644 (160–1 311 264)	145 311 (16–1 962 375)	208 660 (16–2 409 007)	199 228 (32–2 101 537)	145 580 (16–1 512 850)	161 231 (16–2 409 007)

Data represent median (range) unless otherwise indicated. Numbers in brackets indicate number of missing values.

Percentage of total records.

Percentage of age group.

The age threshold used is 12 years. Severe anemia is defined as hemoglobin ≤5 g/dL or hematocrit ≤15% in children <12 years old (<7 g/dL and <20%, respectively, in adults).

More than half of the patients hospitalized with malaria were children ≤15 years of age (54% for *P. vivax* and 57% for *P. falciparum*; *P* = .4). Significantly more infants and young children ≤5 years of age were admitted with *P. vivax* malaria (43%) than with *P. falciparum* malaria (26%) (relative risk [RR], 1.7 [95% CI, 1.5–1.9]; *P* < .001) (Table 1).

In adults (aged >15 years) hospitalized for suspected severe malaria, more women were admitted with *P. vivax* (102 of 128 [80%]) than with *P. falciparum* malaria (650 of 2097 [31%]) (RR, 2.5 [95% CI, 2.3–2.9]; *P* < .001) (Table 1). Pregnant and post-partum women comprised more than half of the women admitted for *P. vivax* (58 of 102 [57%]), and significantly less (123 of 650 [19%]) for *P. falciparum*. Many of the post-partum *P. vivax* patients admitted had reasons for admission that were unrelated to malaria. In hospitalized *P. vivax* malaria cases, the presenting mean hematocrit was 4.2% lower in pregnant women (95% CI, 1.6%–6.9%; *P* = .002) and 7.3% lower in post-partum women (95% CI 3.0–11.5; *P* = .001) than for nonpregnant or non post-partum patients. Chronic disease (including malnutrition, hypertension, cirrhosis, and alcohol dependence) was much more common in patients hospitalized with *P. vivax* than in those hospitalized with *P. falciparum* (RR, 6.8 [95% CI, 2.9–16.1]; *P* < .001) (Table 2). Patients with *P. vivax* malaria were hospitalized for a shorter period (median [interquartile range], 3 [2–4] days) than those with *P. falciparum* (4 [4–6] days); *P* < .001) (Table 2).

**Table 2.** Characteristics of Hospitalized Malaria Patients Who Did or Did Not Meet World Health Organization (2015) Broad Severe Malaria Criteria

	*Plasmodium vivax*			*Plasmodium falciparum*		
Clinical Characteristic	Patients, No. (%)^a^		*P* Value	Patients, No. (%)^a^		*P* Value
	WHO Criteria Not Met (n = 210)	WHO Criteria Met (n = 68)		WHO Criteria Not Met (n = 3362)	WHO Criteria Met (n = 1482)	
Infectious diagnosis present	50 (24)	20 (29)	.35	137 (4)	110 (7)	<.001
Chronic disease present	4 (2)	3 (4)	.25	8 (0.2)	10 (0.7)	.02
Post-partum women^b^	10/127 (8)	7/46 (15)	.16	5/1215 (0.4)	3/599 (0.5)	.72
Prostration	25 (12)	16 (24)	.02	355 (11)	469 (32)	<.001
Convulsions^c^	0 (0)	4 (6)	.003	0 (0)	33 (2)	<.001
Age, y						
<1	48 (23)	8 (12)	.10^d^	55 (2)	35 (2)	<.001^d^
1–5	48 (23)	16 (24)		709 (21)	447 (30)	
6–10	14 (7)	6 (9)		616 (18)	272 (18)	
11–15	8 (4)	2 (3)		473 (14)	140 (9)	
>15	92 (44)	35 (53)		1509 (45)	588 (40)	
Parasitemia, geometric mean (95% CI), parasites/µL	2576 (32–46 472)	1576 (16–31 149)	.12	147 606 (3768–425 030)	196 986 (2304–976 162)	<.001
Duration of hospital stay, median (IQR; range) d	3 (2–4; 1–32)	2 (2–4; 1–26)	.90	4 (3–6; 1–34)	5 (4–6; 1–31)	<.001
Intravenous artesunate	31 (15)	13 (19)	.39	655 (19)	677 (46)	<.001
Blood transfusion	26 (12)	19 (28)	.002	323 (10)	317 (21)	<.001

Abbreviations: CI, confidence interval; IQR, interquartile range; WHO, World Health Organization.

Data represent no. (%) of patients unless otherwise indicated.

The denominators for post-partum women represent the number of female patients aged >15 years.

The WHO 2015 malaria guidelines [25] criterion for convulsions is >2 convulsions in 24 hours. It does not specify that convulsions must be accompanied by a low Glasgow Coma Scale score.

Univariable ordered logistic regression analysis was used to determine the relationship between age groups and whether or not WHO criteria were met.

### Deaths

There were 70 deaths in total, 66 with *P. falciparum* and 4 with *P. vivax* infection. One *P. vivax* malaria death occurred in a 54-year old man with Gram-negative meningitis; *Escherichia coli* grew in an immediate post-mortem cerebrospinal fluid culture. The parasite density was 1 in 500 white blood cells (16/µL). This suggests that the parasitemia was incidental to the fatal bacterial meningitis. Two other *P. vivax* deaths occurred in patients with low parasite densities (<3300/µL), leukocytosis, and a clinical diagnosis of sepsis, although blood cultures were negative. Both patients were anemic and required blood transfusions. The fourth *P. vivax* malaria death occurred in a malnourished woman (HIV and tuberculosis negative) in the third trimester of pregnancy [27]. She experienced acute respiratory distress 67 hours after starting treatment with artesunate-mefloquine [28]. The overall mortality rate in patients hospitalized for malaria was 4 of 278 (1.4%) for *P. vivax* and 66 of 4844 (1.4%) for *P. falciparum* malaria, although only 1 of the *P. vivax* deaths was clearly related to malaria. Analyzed as a proportion of total outpatient confirmed malaria cases, the risk of death associated with symptomatic *P. falciparum* malaria was therefore ≥14 times greater (95% CI, 5.1–38.7) than for *P. vivax* malaria (Figure 2).

### Severe Malaria Admissions

Fewer hospitalized patients with *P. vivax* (68 of 278 [24%]) than with *P. falciparum* (1482 of 4844 [31%]) met the WHO criteria for severe malaria (Table 2 and Supplementary Table 1). Post-partum and pregnant women comprised 55% (6 of 11) of the severe anemia cases with *P. vivax* malaria and 16% (10 of 64) of those with *P. falciparum* malaria (RR, 5.7 [95% CI, 2.0–16.7]; *P* = .001) (Table 3). Impaired consciousness was present in 13% (9 of 68) of the *P. vivax* cases. Pulmonary edema was a more common manifestation of severity in *P. vivax* (27 of 68 [40%]) than in *P. falciparum* malaria (167 of 1482 [11%]) (RR, 3.2 [95% CI, 2.3–4.5]; *P* < .001). Severe anemia was also more common among the patients with severe vivax malaria (18 of 68 [26%]) than among those with severe falciparum malaria (167 of 1482 [11%]) (RR, 1.8 [95% CI, 1.1–2.7]; *P* = .01) (Table 3). The proportions of patients with prostration or convulsions [25] were similar between *P. vivax* and *P. falciparum* malaria. (Supplementary Table 1).

**Table 3.** Hospitalized Malaria Cases Classified as Severe by the World Health Organization (2015) Broad Severe Malaria Criteria

Clinical Characteristic^a^	Patients, No. (%)					
	*Plasmodium vivax*			*Plasmodium falciparum*		
	Age ≤15 y (n = 32)	Age >15 y (n = 36)	Total (n = 68)	Age ≤15 y (n = 894)	Age >15 y (n = 588)	Total (n = 1482)
Impaired consciousness	7 (22)	2 (6)	9 (13)	66 (7)	117 (20)	183 (12)
Prostration	7 (22)	9 (26)	16 (24)	220 (25)	249 (42)	469 (32)
Convulsions	1 (3)	3 (8)	4 (6)	23 (3)	10 (2)	33 (2)
Shock	0	4 (11)	4 (11)	6 (4)	28 (5)	34 (5)
Jaundice^b^	0	0	0	29 (3)	82 (15)	111 (8)
Significant bleeding	0	0	0	0	0	0
Pulmonary edema	14 (44)	13 (36)	27 (40)	123 (14)	44 (7)	167 (11)
Hypoglycemia	2 (15)	2 (11)	4 (13)	41 (6)	15 (4)	56 (5)
Metabolic acidosis^c^	NA	NA	NA	NA	NA	NA
Severe anemia^b,d^	7 (22)	11 (31)	18 (26)	103 (12)	64 (11)	167 (11)
Renal impairment^c^	0	0	0	1 (0.1)	7 (1)	8 (0.5)
Hyperparasitemia	NA	NA	NA	607 (68)	277 (47)	884 (60)

Abbreviation: NA, not available.

Nineteen cases with *P. vivax* and 601 with *P. falciparum* met ≥2 World Health Organization severity criteria [25].

For *P. vivax,* parasite density thresholds were not used in the severity definitions of jaundice or anemia.

Laboratory testing was not available. For renal impairment, the discharge diagnosis was used as a proxy.

Severe anemia is defined as hemoglobin ≤5 g/dL or hematocrit ≤15% in children <12 years old (<7 g/dL and <20%, respectively, in adults).

Applying the stricter research criteria for severe malaria [26], the proportions of *P. vivax* (63 vs 67 of 278) and *P. falciparum* cases (1463 vs 1482 of 4844) classified as severe malaria were largely unchanged (Supplementary Tables 2 and 3). Based on the broad WHO criteria [25], the overall proportion of severe malaria was 0.84/1000 cases (95% CI, 0.7–1.1) *P. vivax* cases (68 of 80 841) compared with 15.7/1000 cases (14.9–16.5) *P. falciparum* cases (1482 of 94 467). Thus, for outpatients presenting with malaria, the risk of severe malaria was 19 (95% CI, 14.7–23.8) times greater in *P. falciparum* than in *P. vivax* infections (*P* < .001).

## DISCUSSION

Although *P. vivax* malaria was very common along the Thailand-Myanmar border, severe vivax malaria was rare, as it is elsewhere in the Greater Mekong subregion. Nearly all malaria deaths in this region result from *P. falciparum*. The relatively low mortality rate of severe falciparum malaria in this series (4.4%) is explained by the high proportion of cases with prostration or otherwise uncomplicated hyperparasitemia, both of which carry a relatively good prognosis, and prompt treatment with artesunate [26, 29]. Our findings contrast with observations from India and the island of New Guinea, the 2 areas reporting high caseloads of severe *P. vivax* malaria [30–34]. In India, the transmission of *P. vivax* and *P. falciparum* is generally low and unstable, as it is in the Greater Mekong subregion [21, 22].

Symptomatic malaria occurs at all ages, and severe *P. vivax* malaria has been reported extensively [31–34]. A 2021 systematic review of 162 studies from India reported that 29.3% of patients hospitalized with *P. vivax* infections had severe malaria [31]. This is similar to the proportion for *P. falciparum* malaria in our study and considerably exceeds the proportion observed for *P. vivax.* The case-specific mortality rate of acute vivax malaria on the Thailand-Myanmar border is >100 times lower than reported from Bikaner in Northwest India, 6 times lower than reported from Manaus in Brazil [34], and half of that reported from Papua in Indonesia [35] (Table 4). It is unclear whether there are genuinely more severe *P. vivax* cases in India, suggesting greater *P. vivax* virulence there, or unusual susceptibility, or whether there is an ascertainment bias in the diagnoses. In contrast, the island of New Guinea has markedly higher *P. vivax* transmission than in other malaria-endemic areas of the world. Young children are affected particularly, and frequent *P. falciparum* and multiple relapsing *P. vivax* infections result in severe anemia and an increased risk of death [30].

**Table 4.** Comparison of Reported Severe Vivax Malaria Cases From 4 Locations

*Plasmodium vivax* Malaria Outcome	Mae Sot, Thailand (Current Report)	Manaus, Brazil [34]^a^	Bikaner, India [32]^a^	Papua, Indonesia [35]
Total consultations, no.	80 841	10 283	843	293 763
Hospitalizations, no. (%)	278 (0.34)	316 (3)	462 (55)	3495 (1)
WHO severity criteria fulfilled, no. (% of hospitalizations)	67 (24)	40 (12.6)	157 (34)	845 (24)
Mortality rate, deaths/1000 cases	0.05	0.3	6.1	0.12

Abbreviation: WHO, World Health Organization.

The Manaus and Bikaner consultations were at tertiary reference hospitals.

Although some patients with acute *P. vivax* malaria did require hospitalization in our series, the majority (75%) did not have severe malaria, and their prognosis was very good. Pregnancy and the postpartum period were particular risk factors for *P. vivax*–associated severe anemia hospitalizations. This reflects both the cumulative impact of recurrent *P. vivax* malaria and the higher risk of anemia in this population generally [36]. Severe anemia (associated with both species of malaria) and otherwise uncomplicated hyperparasitemia (in *P. falciparum* malaria) both have a relatively good prognosis, provided that there is ready access to diagnosis and treatment (with artesunate) and that blood transfusions can be given [26, 29, 36]. Even with acute pulmonary edema in *P. vivax* malaria (which carries a high mortality rate in *P. falciparum* malaria), all but 1 patient survived [26, 27, 29, 37].

Several factors contribute to the “severe *P. vivax* malaria” reporting differences between malaria regions. These relate to the criteria used, definitions applied, and use of proxy indicators to determine severity. For example, severe thrombocytopenia is often used as a severity criterion and accounts for a large proportion of reported “severe vivax” cases [15, 31]. This is not a severity criterion for falciparum malaria [26, 29]. Many severe *P. vivax* case patients have severe anemia (approximately 20% in a meta-analysis [15] and nearly 30% in the current series). The anemia criterion for severe falciparum malaria requires concomitant parasitemia (parasite density, 10 000/µL) to improve specificity [26], but in many reported severe vivax cases, the associated parasite count is not specified. The jaundice criterion for severe falciparum malaria requires a concomitant parasite density of 100 000/µL, which is very unusual in *P. vivax* malaria, whereas jaundice with any parasite density is defined as severe *P. vivax* malaria [25, 26]. This reduces diagnostic specificity, as some patients with uncomplicated malaria may develop transient cholestatic jaundice.

Other possible contributors to the diagnosis of severe *P. vivax* malaria are preexisting conditions (such as often undiagnosed or unconfirmed chronic diseases or coexisting infections [38]), which may cause or predispose to vital organ dysfunction and for which the malaria parasitemia is coincidental rather than a cause of severe illness or death. The patient with fatal Gram-negative meningitis in this series is a probable example of coincidental *P. vivax* infection. Within malaria-endemic areas, misdiagnosis of severe malaria is very common. It is estimated that approximately one-third of African children with diagnosed severe *P. falciparum* malaria, even in specialist research centers, are misdiagnosed [13]. Other factors, such as being very young or old, pregnant, or post-partum, also increase the likelihood of hospitalization. In older and frail adults, and in those debilitated by chronic diseases, acute malaria illness (caused by any species) can prove fatal.

Antimalarial resistance increases *P. vivax* recurrence rates, especially when anti-relapse treatment is not given, and it thereby increases the prevalence and severity of anemia. Chloroquine resistance may be a contributor to reported severe *P. vivax* malaria in India, although the levels of resistance reported to date in South Asia and the Greater Mekong subregion are low [39].

This study had several limitations. This was a retrospective evaluation, so procedures varied in time and place, and missing inpatient records were not included and outcomes were sometimes not known for patients referred to tertiary hospitals. Full blood cell counts were routinely available only in the latter half of the study period. Results of biochemical investigations were not available. Pulmonary edema could not be confirmed with chest radiography, and pulse oximetry was not routinely available. Investigations to exclude alternative diagnoses limited our ability to differentiate causation between severe *P. vivax* and other common infections.

In conclusion, in northwest Thailand along the Myanmar border, *P. vivax* malaria is common, but severe infections and death resulting from *P. vivax* are rare. Further investigation is needed to determine why there are differences in the apparent proportions of severe *P. vivax* malaria across different geographic regions, transmission settings, and relapse intervals.

## Supplementary Data

Supplementary materials are available at *Clinical Infectious Diseases* online. Consisting of data provided by the authors to benefit the reader, the posted materials are not copyedited and are the sole responsibility of the authors, so questions or comments should be addressed to the corresponding author.

## Supplementary Material

ciad262_Supplementary_Data

## Notes

***Acknowledgments.*** The results from this study reflect more than 30 years of humanitarian field work performed by the Shoklo Malaria Research Unit (SMRU) healthcare providers, medical staff, and the laboratory, data, logistic, and administrative teams. These efforts contribute directly to the SMRU academic outputs. Many thanks to all who supported malaria diagnosis, treatment, and management from 2000 to 2016. The authors especially thank the patients for placing their trust in SMRU.

***Financial support*.** This work is supported by the Wellcome Trust (grant 089179 to C. S. C., R. M., N. J. W., and F. N.). For the purpose of open access, the authors have applied a CC BY public copyright license to any Author Accepted Manuscript version arising from this submission.
