# Severe falciparum and vivax malaria on the Thailand-Myanmar border: A review of 1503 cases

**DOI:** 10.1093/cid/ciad262

**Published:** 2023-05-05

**Authors:** Cindy S Chu, Marie Stolbrink, Daniel Stolady, Makoto Saito, Candy Beau, Kan Choun, Tha Gay Wah, Ne Mu, Klay Htoo, Be Nu, Arunrot Keereevijit, Jacher Wiladpaingern, Verena Carrara, Aung Pyae Phyo, Khin Maung Lwin, Christine Luxemburger, Stephane Proux, Prakaykaew Charunwatthana, Rose McGready, Nicholas J White, François Nosten

**Affiliations:** 1 Shoklo Malaria Research Unit, Mahidol–Oxford Tropical Medicine Research Unit, Faculty of Tropical Medicine, Mahidol University, Mae Sot, Thailand; 2 Centre for Tropical Medicine and Global Health, Nuffield Department of Medicine, University of Oxford, Oxford, United Kingdom; 3 Division of Infectious Diseases, Advanced Clinical Research Center, Institute of Medical Science, University of Tokyo, Tokyo, Japan; 4 Institute of Global Health, Faculty of Medicine, University of Geneva, Geneva, Switzerland; 5 Mahidol–Oxford Tropical Medicine Research Unit, Faculty of Tropical Medicine, Mahidol University, Bangkok, Thailand; 6 Department of Clinical Tropical Medicine, Faculty of Tropical Medicine, Mahidol University, Bangkok, Thailand

**Keywords:** Plasmodium vivax, Plasmodium falciparum, Severe malaria, Epidemiology

## Abstract

**Background:**

The northwestern border of Thailand is an area of low seasonal malaria transmission. Until recent successful malaria elimination activities, malaria was a major cause of morbidity and mortality. Historically the incidences of symptomatic *Plasmodium falciparum* and *Plasmodium vivax* malaria were approximately similar.

**Methods:**

All malaria cases managed in the Shoklo Malaria Research Unit along the Thailand-Myanmar border between 2000 and 2016 were reviewed.

**Results:**

There were 80,841 consultations for symptomatic *P. vivax* and 94,467 for symptomatic *P. falciparum* malaria. Overall 4,844 (5.1%) patients with *P. falciparum* malaria were admitted to field hospitals, of whom 66 died, compared with 278 (0.34%) with *P. vivax* malaria, of whom four died (three were diagnosed with sepsis, so the contribution of malaria to their fatal outcomes is uncertain). Applying the 2015 “World Health Organization severe malaria criteria”, 68/80,841 (0.08%) of *P. vivax* and 1,482/94,467 (1.6%) of *P. falciparum* admissions were classified as severe. Overall, patients with P. falciparum malaria were 15 (95% CI 13.2-16.8) times more likely than *P. vivax* to require hospital admission, 19 (95% CI 14.6-23.8) times more likely to develop severe malaria, and at least 14 (95% CI 5.1-38.7) times more likely to die.

**Conclusions:**

In this area both *P. falciparum* and *P. vivax* infections were important causes of hospitalization, but life-threatening *P. vivax* illness was rare.

## Introduction

Early in the 20th century, *Plasmodium vivax* was known as “benign tertian malaria” to differentiate it from the commonly fatal “malignant tertian” malaria caused by *Plasmodium falciparum* [1]. In the 1920s, early in the era of malaria therapy for neurosyphilis [2,3], the lethal potential of *P. falciparum* was soon evident, and so artificial infection with the safer *P. vivax* malaria became the therapy of choice. Even so, malaria therapy showed that patients who were already severely debilitated by neurosyphilis could die as a result of any of the human malaria infections-even *P. malariae* [4]. It was also recognized that *P. vivax* generally caused fever at lower parasite densities than *P. falciparum* [5]. The early descriptions of malaria from military and malaria therapy experiences which created the “textbook” descriptions referred usually to previously unexposed (i.e. non-immune) adults [6,7]. They are more relevant today to malaria in travelers, and less so to malaria in endemic areas where the population is exposed repeatedly to malaria infections. In low transmission areas malaria occurs at all ages, whereas in higher transmission settings, with the acquisition of disease controlling immunity, malaria illness is largely confined to younger children. It is estimated that over 90% of the deaths from severe malaria in the world are in African children. Nearly all are attributable to *P. falciparum* [8]. In higher transmission settings, where asymptomatic patent malaria parasitemia is very common, distinguishing malaria as the cause of illness from other illnesses with coincidental malaria parasitemia is difficult [9–11]. Even in low transmission areas, a significant proportion of the community has asymptomatic parasitemia [12]. Directly attributable malaria mortality is consequently overestimated-often by a substantial amount [13].

In recent years, as the global burden of malaria and the geographic extent of malaria endemic areas have decreased, the number of reports of severe vivax malaria has increased markedly. It is unclear whether this represents a genuine rise, increased recognition, a lower threshold for the diagnosis, incorrect attribution or selective reporting. The prevalence of severe *P. vivax* infections has varied markedly both across and within geographical regions [14–20]. Clinical manifestations reported in severe *P. vivax* malaria are similar to those reported in severe *P. falciparum* malaria [14]. They include pulmonary edema, severe anemia, shock, hypoglycemia, hepatic or renal dysfunction, neurologic dysfunction (cerebral malaria or multiple convulsions), and severe thrombocytopenia [15]. Extremes of age, co-morbidities, and chloroquine resistance have been associated with an increased risk of severe vivax malaria [17–20]. As in the earlier malaria therapy experience, already debilitated patients are at greatest risk [3,4]. A recent meta-analysis estimated that 1.1% of symptomatic *P. vivax* infections had severe malaria (11,658/1,059,0970 total cases). Case fatality was reported as 5% in patients with at least one severe manifestation [14]. Very few reports have come from the countries of the Greater Mekong sub-region where severe *P. vivax* malaria is considered rare [16]. These marked geographic differences, the lack of detailed prospective clinical studies, and the difficulty in establishing causality leave substantial uncertainty over the true incidence, prevalence, and outcomes of severe vivax malaria. This retrospective review of all patients admitted to clinics and hospitals at the Shoklo Malaria Research Unit (SMRU) on the northwestern border of Thailand over a 16-year period was performed to provide a comparative assessment of prevalence and severity of illness caused by these two main malaria species.

## Methods

### Study population

This observational study was conducted by SMRU, which has operated malaria clinics and inpatient facilities along the northwestern Thailand-Myanmar border since 1986. The epidemiology of malaria in this region of hill forest and low seasonal malaria transmission has been studied in detail and reported previously [21,22]. The patient population comprised migrant workers and displaced persons of all ages of Burman and Karen ethnicities. Until 2012, patient numbers in the transmission season were very high with nearly 200 consultations each day at one health clinic (50% with confirmed malaria). Before 2010, malaria was diagnosed either by a *P. falciparum* specific rapid diagnostic test (RDT) or malaria smear, whereby parasite counts were provided for *P. falciparum*, and *P. vivax* infection was noted but not quantitated. After 2010, P. vivax parasite densities were quantitated. All patients with >4% *P. falciparum* parasitemia (hyperparasitawere admitted. Otherwise patients were hospitalized at physician discretion. All had a malaria smear performed. Pulse oximetry was not available before 2009, and until 2015 was not performed routinely. Three days of oral chloroquine (25mg base/kg) was given for *P. vivax* malaria, and 3 days of oral artemisinin combination therapy (ACT) (e.g., mefloquine-artesunate, artemether-lumefantrine, dihydroartemisinin-piperaquine) was given for *P. falciparum* malaria. As second line, or for treatment failure, in *P. falciparum* malaria, 7-days of quinine or artesunate combined with doxycycline or clindamycin was given [23]. Primaquine radical cure for *P. vivax* was not prescribed routinely during the study period. Patients with >4% parasitemia were given artesunate (oral or intravenous depending on clinical condition), and completed 7 days treatment with an ACT [23]. Other medical management included anti-convulsants to treat seizures, blood transfusions, oral or intravenous antibiotics, and intravenous fluids. Positive pressure ventilation and renal replacement therapies were not available. Full blood counts, biochemical and microbiology investigations were not available routinely before 2010.

### Study methods

All patients attending SMRU outpatient clinics had an electronic data entry. Anonymized data from October 2000 to December 2016 provided the total number of outpatient consultations with a malaria diagnosis. For the same period, records of patients admitted to the SMRU hospitals with *P. falciparum* or *P. vivax* malaria were obtained from the inpatient electronic database [24]. To account for hospitalizations for post-delivery complications unrelated to malaria, post-partum women were defined as those from one calendar day after and up to <6 weeks from the date of delivery. Dates, age, sex, weight, medical history, presenting symptoms and their duration, vital signs, clinical examination, results of diagnostic tests performed, treatment and discharge diagnoses were extracted. Severe malaria diagnoses were based on the current broad WHO classification [25] and also analyzed using the stricter research definition which excludes prostration or convulsions as severe malaria criteria [26] (Supplementary Table 1). Severe anaemia was defined as in the WHO classification; hemoglobin ≤5g/dL or hematocrit ≤15% in children <12 years (<7g/dL and <20% respectively in adults). Chest radiography was not available on site. Pulmonary edema was diagnosed clinically if pulse oximetry was <92% on room air OR, if oximetry was unavailable, if the respiratory rate was elevated for age AND chest examination was abnormal. If the patient was visibly jaundiced the serum total bilirubin was assumed to be >50μmol/L (>3mg/dL). If a discharge diagnosis of renal failure was recorded, the patient hospital record was reviewed to determine the basis for the diagnosis. Parasite density thresholds for *P. vivax* were not used in the severity definitions [26].

### Statistical analysis

Comparisons were made using Chi-squared or Fisher’s exact or Student’s t or non-parametric K-sample tests as appropriate. Multivariable generalized linear modelling was used to assess the effects of age, sex, pregnancy and postpartum status, malaria species, the presence of concomitant disease, on whether the WHO severe malaria criteria were met [25,26] for *P. vivax* compared to *P. falciparum*. Statistical analysis was performed using Stata 15.1 (StataCorp LP).

### Ethics review

Ethics approval was given by the Ethics Committee at the Faculty of Tropical Medicine, Mahidol University (TMEC 17-049), Oxford Tropical Research Ethics Committee (OXTREC 28-09) and the Tak Community Advisory Board (20170729/TCAB-11).

## Results

**Figure 1:**
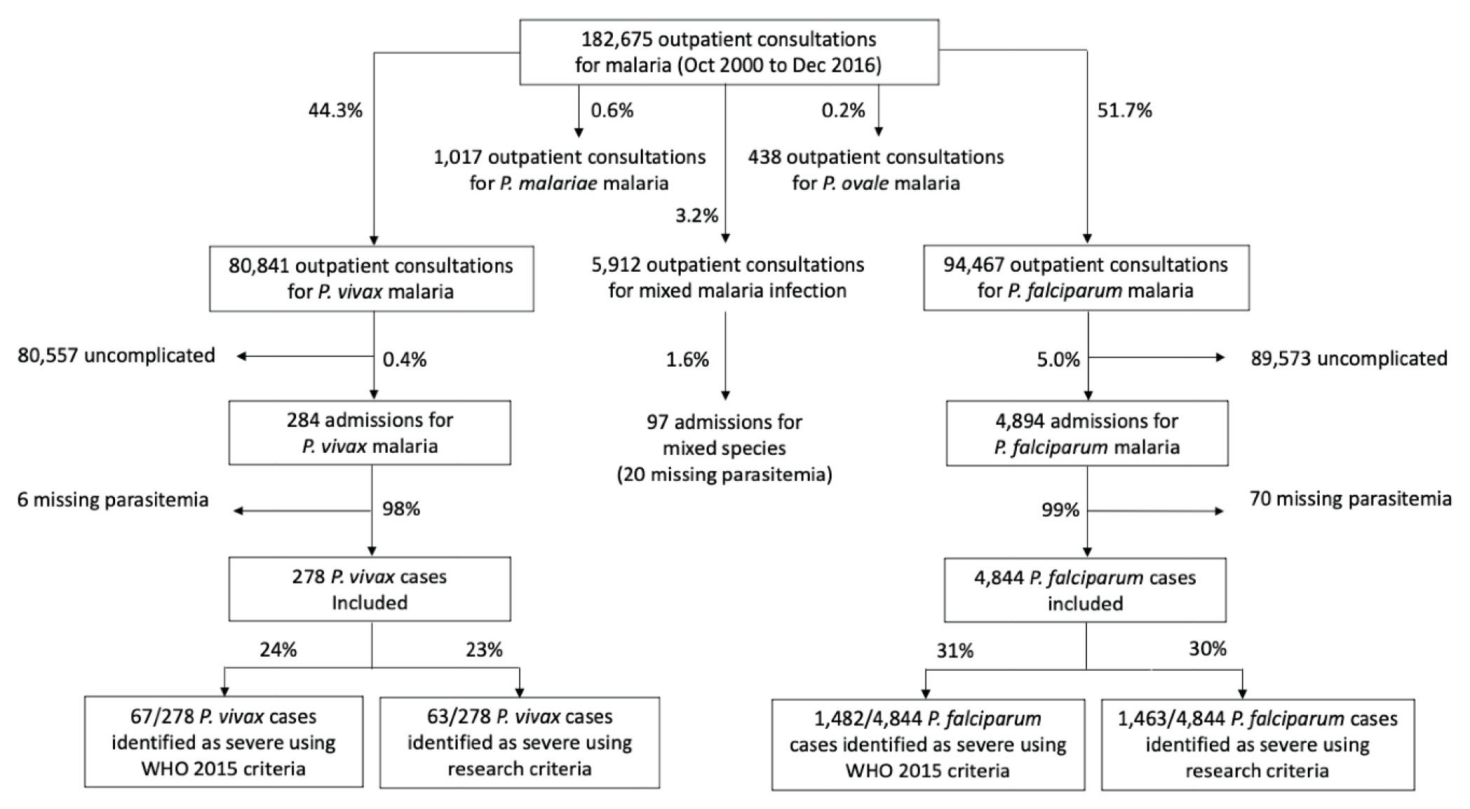
Flow diagram of malaria cases included. Excluded cases are not enclosed in an outlined box.

**Figure 2:**
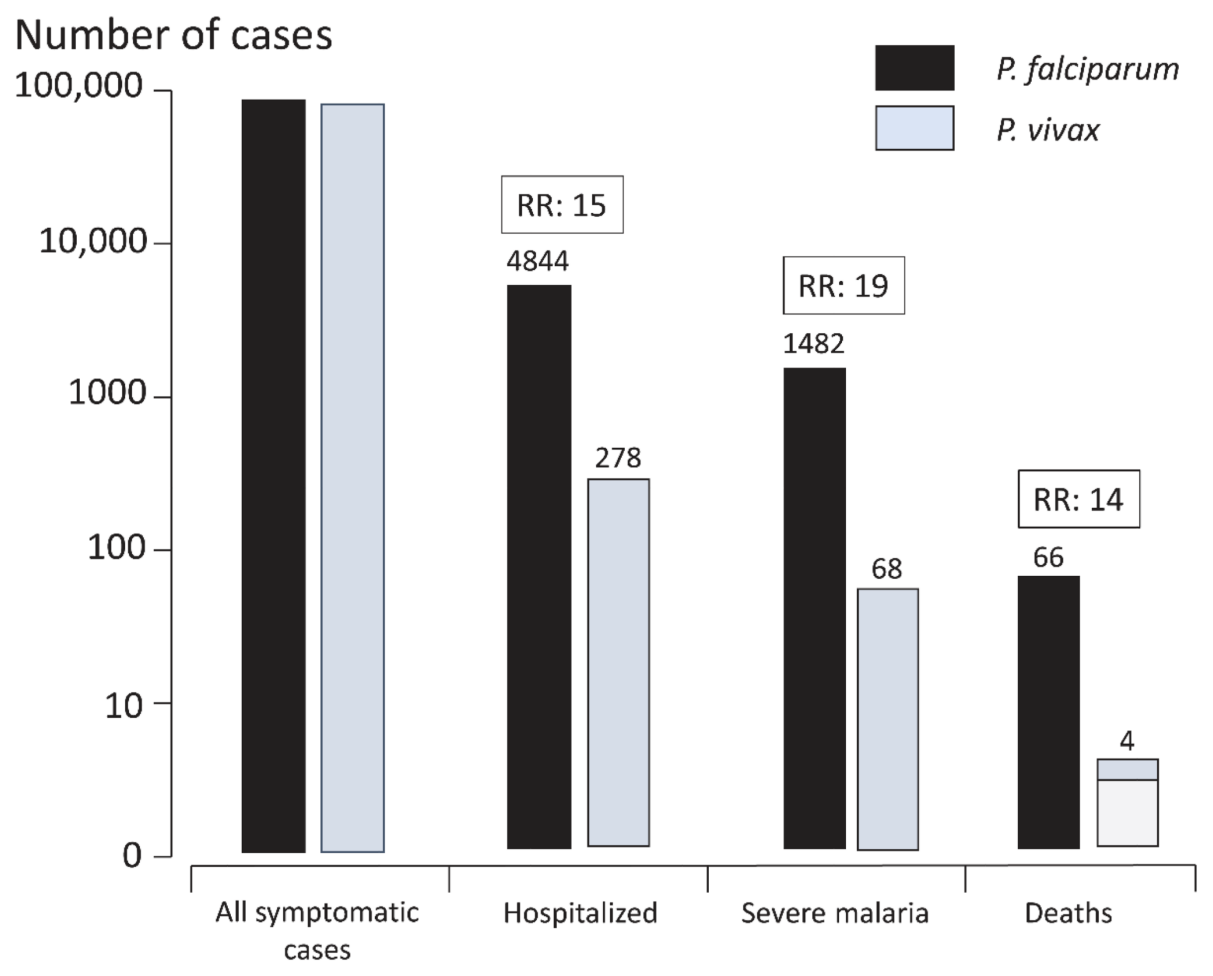
The relative risk of hospitalization, severe malaria, and death for *Plasmodium falciparum* compared to *Plasmodium vivax*. In three of the four fatalities with *P. vivax* malaria (light grey box) the primary diagnosis was sepsis and the causal role of malaria was uncertain.

Between October 2000 and December 2016, there were 80,841 consultations for *P. vivax* malaria, 94,467 for *P. falciparum*, 1,017 for *Plasmodium malariae*, and 438 for *Plasmodium ovale*. The vast majority of patients (*P. vivax*: 80,557; 99.6% and *P. falciparum*: 89,573; 95%), had uncomplicated illness. Mixed species infections were documented in 3.2% (5,912) of the 182,675 symptomatic malaria infections. 175,308 cases were included in this study; 278 (0.34%) were hospitalized with a diagnosis of severe *P. vivax* malaria, and 4,844 (5.0%) were hospitalized for severe *P. falciparum* malaria (Figure 1). Thus, symptomatic *P. falciparum* infections were 15 (95% CI 13.2-16.8) times more likely than *P. vivax* infections to require hospital admission, p<0.001 (Figure 2). Parasite densities in patients hospitalized with *P. vivax* infections ranged from 16 to 189,028/μL (geometric mean 2,284/μL) and for *P. falciparum* ranged from 16 to 2,409,007/μL (geometric mean 161,231/μL) (Table 1). Over half the hospitalized malaria patients were children ≤15 years of age (*P. vivax*: 54% and *P. falciparum*: 57%, p=0.4). Significantly more infants and young children ≤5 years of age were admitted with *P. vivax* malaria (43%) compared to *P. falciparum* malaria (26%), RR 1.7, 95% CI 1.5-1.9; p<0.001 (Table 1). In adults (>15yr) hospitalized for suspected severe malaria, more females were admitted with *P. vivax* (102/128, 80%) than *P. falciparum* malaria (650/2,097, 31%), RR 2.5, 95% CI 2.3-2.9; p<0.001 (Table 1). Pregnant and postpartum females comprised over half of the females admitted for *P. vivax* (58/102; 57%), and significantly less (123/650; 19%) for *P. falciparum*. Many of the post-partum *P. vivax* patients admitted had reasons for admission that were unrelated to malaria. In hospitalized *P. vivax* malaria cases, presenting mean hematocrit was 4.2% lower in pregnant women (95% CI 1.6-6.9; p=0.002) and 7.3% lower in postpartum women (95% CI 3.0-11.5; p=0.001) than for non-pregnant or non-postpartum patients. Chronic disease (including malnutrition, hypertension, cirrhosis, and alcohol dependence) was much more common in hospitalized *P. vivax* than *P. falciparum* cases, RR 6.8, 95% CI 2.9-16.1; p<0.001 (Table 2). *P. vivax* malaria patients were hospitalized for a shorter period (median 3 days, IQR 2-4) than *P. falciparum* cases (median 4 days, IQR 4-6); p<0.001 (Table 2).

### Deaths

There were 70 deaths in total; 66 *P. falciparum* and 4 *P. vivax* infections. One *P. vivax* malaria death occurred in a 54-year old male with Gram-negative meningitis; an immediate post-mortem cerebrospinal fluid culture grew *Escherichia coli*. The parasite density was 1 in 500 WBC (16/μL). This suggests that the parasitemia was incidental to the fatal bacterial meningitis. Two other *P. vivax* deaths occurred in patients with low parasite densities (<3300/μL), leukocytosis and a clinical diagnosis of sepsis, although blood cultures were negative. Both were anemic and required blood transfusions. The fourth *P. vivax* malaria death occurred in a malnourished woman (HIV and TB negative) in the 3^rd^ trimester of pregnancy [27]. She developed acute respiratory distress 67 hours after starting treatment with artesunate-mefloquine [28]. The overall mortality in hospitalized cases with *P. vivax* malaria was 4/278 (1.4%), and for *P. falciparum* malaria was 66/4,844 (1.4%), although only one of the *P. vivax* deaths was clearly related to malaria. Analyzed as a proportion of total outpatient confirmed malaria cases, the risk of death associated with symptomatic *P. falciparum* malaria was therefore at least 14 times greater (95% CI 5.1-38.7) than for *P. vivax* malaria (Figure 2).

### Severe malaria admissions

Fewer hospitalized *P. vivax* (68/278, 24%) than *P. falciparum* cases (1,482/4,844, 31%) met the WHO criteria for severe malaria (Table 2 and Supplementary Table 1). Postpartum and pregnant women comprised 55% (6/11) of the severe anemia cases with *P. vivax* malaria and 16% (10/64) of those with *P. falciparum* malaria, RR 5.7, 95% CI 2.0-16.7; p=0.001 (Table 3). Impaired consciousness was present in 13% (9/68) of the *P. vivax* cases. Pulmonary edema was a more common manifestation of severity in *P. vivax* (27/68; 40%) than in *P. falciparum* malaria (167/1,482, 11%), RR 3.2, 95% CI 2.3-4.5; p<0.001. Severe anemia was also more common among the “severe vivax” (18/68, 26%) compared with the “severe falciparum” patients: 167/1,482, (11%), RR 1.8, 95% CI 1.1-2.7; p=0.01 (Table 3). The proportion of cases with prostration or convulsions [25] was similar between *P. vivax* and *P. falciparum* malaria. (Supplementary Table 1). Applying the stricter research criteria for severe malaria [26], the proportions of *P. vivax* (63 versus 67/278) and *P. falciparum* cases (1,463 versus 1,482/4,844) classified as severe malaria were largely unchanged (Supplementary Tables 2 and 3). Based on the broad WHO criteria [25], the overall proportion of severe *P. vivax* malaria was 0.84/1000 *P. vivax* cases (68/80,841; 95% CI 0.7-1.1) compared with 15.7/1000 *P. falciparum* cases (1,482/94,467; 95% CI 14.9–16.5). Thus, for outpatients presenting with malaria the risk of severe malaria was 19 (95% CI 14.7-23.8) times greater in *P. falciparum* than in *P. vivax* infections; P<0.001.

## Discussion

Although *Plasmodium vivax* malaria was very common along the Thailand-Myanmar border, severe vivax malaria was rare, as it is elsewhere in the Greater Mekong sub-region (GMS). Nearly all malaria deaths in this region result from *P. falciparum*. The relatively low mortality of severe falciparum malaria in this series (4.4%) is explained by the high proportion of cases with prostration or otherwise uncomplicated hyperparasitemia, both of which carry a relatively good prognosis, and prompt treatment with artesunate [26, 29]. Our findings contrast with observations from India and the island of New Guinea, the two areas reporting high caseloads of severe P. vivax malaria [30–34]. In India, the transmission of *P. vivax* and *P. falciparum* is generally low and unstable, as it is in the GMS [21,22]. Symptomatic malaria occurs at all ages, and severe *P. vivax* malaria has been reported extensively [31–34]. A recent systematic review of 162 studies from India reported that 29.3% of patients hospitalised with *P. vivax* infections had severe malaria [31]. This is similar to the proportion for *P. falciparum* malaria in our study and considerably exceeds the proportion observed for *P. vivax*. The case specific mortality of acute vivax malaria on the Thailand-Myanmar border is over one hundred times lower than reported from Bikaner in Northwest India, six times lower than reported from Manaus in Brazil [34], and half of that reported from Papua in Indonesia [35] (Table 4). It is unclear whether there are genuinely more severe *P. vivax* cases in India, suggesting greater *P. vivax* virulence there, or unusual susceptibility, or whether there is an ascertainment bias in the diagnoses. In contrast, the island of New Guinea has markedly higher *P. vivax* transmission than in other malaria endemic areas of the world. Young children are affected particularly and frequent *P. falciparum* and multiple relapsing *P. vivax* infections result in severe anemia and an increased risk of death [30].

Although some patients with acute *P. vivax* malaria did require hospitalization in our series, the majority (75%) did not have severe malaria, and their prognosis was very good. Pregnancy and the post-partum period were particular risk factors for *P. vivax* associated severe anemia hospitalizations. This reflects both the cumulative impact of recurrent *P. vivax* malaria and the higher risk of anemia in this population generally [36]. Severe anemia (associated with both species of malaria) and otherwise uncomplicated hyperparasitemia (in *P. falciparum* malaria) both have a relatively good prognosis provided there is ready access to diagnosis and treatment (with artesunate), and blood transfusions can be given [26, 29, 36]. Even with acute pulmonary edema in *P. vivax* malaria (which carries a high mortality in *P. falciparum* malaria), all but one patient survived [26,27, 29, 37].

Several factors contribute to the “severe *P. vivax* malaria” reporting differences between malaria regions. These relate to the criteria used, definitions applied, and use of proxy indicators to determine severity. For example, severe thrombocytopenia is often used as a severity criterion and accounts for a large proportion of reported “severe vivax” cases [15,31]. This is not a severity criterion for falciparum malaria [26,29]. Many severe *P. vivax* cases have severe anemia (approximately 20% in a recent meta-analysis [15], and nearly 30% in this series). The anemia criterion for severe falciparum malaria requires a concomitant parasitemia of 10,000 parasites/μL to improve specificity [26], but in many reported “severe vivax” cases, the associated parasite count is not specified. The jaundice criterion for severe falciparum malaria requires a concomitant parasite density of 100,000/μL, which is very unusual in *P. vivax* malaria, whereas jaundice with any parasite density is defined as severe *P. vivax* malaria [25,26]. This reduces diagnostic specificity as some patients with uncomplicated malaria may develop transient cholestatic jaundice. Other possible contributors to the diagnosis of severe *P. vivax* malaria are pre-existing conditions (such as often undiagnosed or unconfirmed chronic diseases or co-existing infections [38] which may cause or predispose to vital organ dysfunction, and where the malaria parasitemia is coincidental rather than a cause of severe illness or death. The patient with fatal Gram-negative meningitis in this series is a probable example of coincidental *P. vivax* infection. Within malaria endemic areas misdiagnosis of severe malaria is very common. It is estimated that approximately one third of African children diagnosed with severe *P. falciparum* malaria, even in specialist research centers, are misdiagnosed [13]. Other factors such as being very young or old, or pregnant or postpartum also increase the likelihood of hospitalization. In the elderly and frail, and in those debilitated with chronic diseases, acute malaria illness (caused by any species) can prove fatal.

Antimalarial resistance increases *P. vivax* recurrence rates, especially when anti-relapse treatment is not given, and thereby increases the prevalence and severity of anemia. Chloroquine resistance may be a contributor to reported severe *P. vivax* malaria in India, although the levels of resistance reported to date in South Asia and the Greater Mekong subregion are low [39].

There were several limitations to this study. This was a retrospective evaluation, so procedures varied in time and place, and missing inpatient records were not included and outcomes were sometimes not known for patients referred to tertiary hospitals. Full blood counts were only available routinely in the latter half of the study period. Biochemical investigations were not available. Pulmonary edema could not be confirmed with chest radiography and pulse oximetry was not available routinely. Investigations to exclude alternative diagnoses limited our ability to differentiate causation between severe *P. vivax* and other common infections.

## Conclusions

In northwest Thailand along the Myanmar border, *P. vivax* malaria is common but severe infections and death resulting from *P. vivax* are rare. Further investigation is needed to determine why there are differences in the apparent proportions of severe *P. vivax* malaria across different geographical regions, transmission settings, and relapse intervals.

## Supplementary Material

Supplementary

## Figures and Tables

**Table 1:** Characteristics of patients admitted to SMRU hospitals between 2000 and 2016 with *Plasmodium vivax* and *Plasmodium falciparum* malaria

Plasmodium vivax	Age group						
Characteristic	0-28 days	29 days to <1 year	1-5 years	6-10 years	11-15 years	>15 years	Total
All records, n (%^a^)	11 (4)	45(16)	64(23)	20 (7)	10 (4)	128 (46)	278 (100)
Males, n (%^b^)	5 (45)	24 (53)	33 (52)	11 (55)	6 (60)	26 (20)	105 (38)
Weight, kg	3.4 (1.5 to 4.4)	1 6.5 (2.6 to 9.7)	10.0 (6.3 to 18)1	1 16.5 (11 to 28)	32.5 (25 to 49)	49 (30 to 71)^1^	26 (1.5 to 71)^3^
Temperature, °C	38.5 (36.5 to 39.8)	37.8 (36.3 to 40.0)	38.8 (36.5 to 40.7)	38.0 (36.0 to 40.3)	38.2 (37.4 to 40.1)	38.0 (35.9 to 41.2)	38.2 (35.9 to 41.2)
Heart rate, bpm	148 (90 to 180)	140 (90 to 188)	131 (98 to 199)	126 (84 to 160)	98 (72 to 120)	96 (66 to 148)	119 (66 to 199)
Respiratory rate, per minute	48(30 to 80)	46(20 to 76)	39(20 to 90)	32(22 to 48)	26(20 to 38)	28(14 to 52)	32(14 to 90)
Hematocrit, %	36 (29 to 47)^2^	27 (12 to 50)^2^	30 (9 to 44)^2^	29 (11 to 40)^4^	33 (12 to 43)^2^	33 (3 to 56)^8^	32 (3 to 56)^20^
Records meeting severe anaemia definition^c^, n (%^b^)	0	2 (4)	2 (3)	2 (10)	1 (10)^c^	11 (9)^c^	18 (6)
Parasitemia/¼L, geometric mean (range)	9,871(256 to 189,028)	1,738(16 to 125,600)	2,719(16 to 143,184)	1,350(48 to 58,027)	1,713(32 to 35,168)	2,257(16 to 105,504)	2,284(16 to 189,028)
**Plasmodium falciparum**	Age group					
Characteristic	0-28 days	29 days to <1 year	1-5 years	6-10 years	11-15 years	>15 years	Total
All records, n (%^a^)	2 (0.04)	88 (2)	1,156 (24)	888 (18)	613 (13)	2,097 (43)	4,844 (100)
Males, n (%^b^)	1 (50)	43 (49)	615 (53)	520 (59)	404 (66)	1, 447 (69)	3,030 (63)
Weight kg	2.5 (2.0 to 2.9)	7.0 (2.6 to 11)	12 (5.0 to 25)^1^	19 (8 to 50)	33 (16 to 58)^1^	50 (20 to 84)^6^	31 (2 to 84)^8^
Temperature, °C	36.9 (35.8 to 38.0)	37.8 (34.8 to 40.3)	38.1 (35.5 to 40.9)	38.2 (35.5 to 41.5)	38.1 (35.0 to 41.5)	38.0 (34.7 to 41.6)^3^	38.0 (34.7 to 41.6)^3^
Heart rate, bpm	139 (120 to 158)	140 (100 to 190)	132 (60 to 200)	120 (64 to 200)	110 (64 to 160)^1^	100 (60 to 160)^35^	114 (60 to 200)^36^
Respiratory rate, per minute	39 (34 to 44)	44 (26 to 90)	36 (14 to 80)	30 (14 to 60)	28 (18 to 52)^1^	26 (10 to 62)^35^	28 (10 to 90)^36^
Hematocrit, %	40 (26 to 54)	26 (9 to 45)	29 (6 to 61)^9^	33 (9 to 50)^5^	36 (8 to 54)^1^	37 (5 to 77)^23^	34 (5 to 77)^38^
Met the severe anaemia definition^c^, n (%^b^)	0	12 (14)	64 (6)	18 (2)	9 (1)^c^	64 (3)^c^	167 (3)
Parasitemia/μL, geometric mean (range)	9,655 (128 to 728,229)	124,644 (160 to 1,311,264)	145,311 (16 to 1,962,375)	208,660 (16 to 2,409,007)	199,228 (32 to 2,101,537)	145,580 (16 to 1,512,850)	161,231 (16 to 2,409,007)

Data are presented at median (range) unless otherwise indicated Numeric superscripts indicate number of missing values

a per cent is of the total records

b per cent is of the age group

c Age threshold used is 12 years. Severe anemia is defined as hemoglobin ≤5g/dL or hematocrit ≤15% in children <12 years (<7g/dL and <20% respectively in adults)

**Table 2:** Characteristics of hospitalized malaria patients who did, or did not meet WHO (2015) broad severe malaria criteria

	*Plasmodium vivax*			*Plasmodium falciparum*	
Clinical characteristics	WHO 2015 criteria not met	WHO 2015 criteria met	p value	WHO 2015 criteria not met	WHO 2015 criteria met	p value
	N=210, n (%)	N=68, n (%)		N=3,362, n (%)	N=1,482, n (%)	
Infectious diagnosis present	50 (24)	20 (29)	0.35	137 (4)	110 (7)	<0.001
Chronic disease present	4 (2)	3 (4)	0.25	8 (0.2)	10 (0.7)	0.02
Postpartum females^a^	10/127 (8)	7/46 (15)	0.16	5/1,215 (0.4)	3/599 (0.5)	0.72
Prostration	25 (12)	16 (24)	0.02	355 (11)	469 (32)	<0.001
Convulsions^b^	0 (0)	4 (6)	0.003	0 (0)	33 (2)	<0.001
Age <1 year	48 (23)	8 (12)		55 (2)	35 (2	
Age 1-5 years	48 (23)	16 (24)		709 (21)	447 (30)	
Age 6-10 years	14 (7)	6 (9)	0.10^c^	616 (18)	272 (18)	<0.001^c^
Age 11-15 years	8 (4)	2 (3)		473 (14)	140 (9)	
Age >15 years	92 (44)	35 (53)		1,509 (45)	588 (40)	
Parasitemia/μL, Geometric mean (95% CI)	2,576 (32-46,472)	1,576 (16-31,149)	0.12	147,606 (3,768-425,030)	196,986 (2,304-976,162)	<0.001
Duration of hospital stay, median (IQR, range) days	3 (2-4, 1-32)	2 (2-4, 1-26)	0.90	4 (3-6, 1-34)	5 (4-6, 1-31)	<0.001
Intravenous artesunate	31 (15)	13 (19)	0.39	655 (19)	677 (46)	<0.001
Blood transfusion	26 (12)	19 (28)	0.002	323 (10)	317 (21)	<0.001

Data are presented at median (range) unless otherwise indicated

a The denominator is the number of females <15 years.

b The WHO 2015 malaria guidelines [25] criterion for convulsions is more than 2 convulsions in 24 hours. It does not specify that convulsions must be accompanied by low Glasgow Coma Score.

c Univariable ordered logistic regression analysis was used to determine relationship between age groups and whether or not WHO criteria were met.

**Table 3:** Hospitalized malaria cases classified as severe by the WHO (2015) broad severe malaria criteria [25]

	*Plasmodium vivax*			*Plasmodium falciparum*	
Clinical characteristic^a^	Age ≤15 years	Age >15 years	Total	Age ≤15 years	Age >15 years	Total
	N=32, n (%)	N=36, n (%)	N=68, n (%)	N=894, n (%)	N=588, n (%)	N=1,482, n (%)
Impaired consciousness^a^	7 (22)	2 (6)	9 (13)	66 (7)	117 (20)	183 (12)
Prostrate	7 (22)	9 (26)	16 (24)	220 (25)	249 (42)	469 (32)
Convulsions	1 (3)	3 (8)	4 (6)	23 (3)	10 (2)	33 (2)
Shock	0	4 (11)	4 (11)	6 (4)	28 (5)	34 (5)
Jaundice^b^	0 (0)	0 (0)	0 (0)	29 (3)	82 (15)	111 (8)
Significant bleeding	0 (0)	0 (0)	0 (0)	0 (0)	0 (0)	0 (0)
Pulmonary edema	14 (44)	13 (36)	27 (40)	123 (14)	44 (7)	167 (11)
Hypoglycemia	2 (15)	2 (11)	4 (13)	41 (6)	15 (4)	56 (5)
Metabolic acidosis_c_	NA	NA	NA	NA	NA	NA
Severe anemia^b,d^	7 (22)	11 (31)	18 (26	103 (12)	64 (11)	167 (11)
Renal impairment^c^	0	0	0	1 (0.1)	7 (1)	8 (0.5)
Hyperparasitemia	NA	NA	NA	607 (68)	277 (47)	884 (60)

a 19 cases with *P. vivax* and 601 cases with *P. falciparum* met at least two WHO severe criteria.

b For *P. vivax* parasite density thresholds were not used in the severity definitions of jaundice or anemia.

c Laboratory testing was not available. For renal impairment the discharge diagnosis was used as a proxy.

d Severe anemia is defined as hemoglobin ≤5g/dL or hematocrit ≤15% in children <12 years, and <7g/dL and <20% respectively in adults.

**Table 4:** A comparison of reported severe vivax malaria from three locations. Compares the current report with references 34 and 35.

	Mae Sot (Thailand)	Manaus (Brazil)	Bikaner (India)	Papua (Indonesia)
Consultations with *P. vivax* malaria	80,841	10,283	843	293,763
Hospitalised with *P. vivax* malaria	278 (0.34%)	316 (3%)	462 (55%)	3,495 (1%)
Fulfilled WHO severity criteria	67 (24%)	40 (12.6%)	157 (34%)	845 (24%)
Mortality per 1000 cases	0.05	0.3	6.1	0.12

The Manaus and Bikaner consultations were at tertiary reference hospitals.
