# Supplementary material for: Severe falciparum and vivax malaria on the Thailand-Myanmar border: A review of 1503 cases
Source: Clin Infect Dis. Author manuscript; Available in PMC 2023 Sep 12. (PMC10495127; doi:10.1093/cid/ciad262)
Supplement: Supplementary [file EMS175249-supplement-Supplementary.doc]

**Supplementary Table 1.**

WHO 2015 severe malaria criteria

| **Severe**  ***P. falciparum* malaria criterion** | **How the clinical record was assessed against the WHO criteria** | **Analysis using the WHO**  **(2015) broad definition**  **for severe *P. falciparum***  **malaria (25)** | **Analysis using the WHO (2014) research definition for severe *P. falciparum* malaria (26)** |
| --- | --- | --- | --- |
| Impaired consciousness | GCS and BCS assessment at admission | Glasgow coma score (GCS) <11 in adults or Blantyre coma score <3 in children | same |
| Prostration | Clinical examination at admission | Unable to walk or sit without assistance | removed |
| Convulsions | Number of convulsions in the clinical history, and during admission and hospitalization | >2 convulsions in 24 hours | removed |
| Shock | Systolic blood pressure (SBP) measurement at admission | SBP <80mmHg in adults or <70mmHg in children. | same |
| Jaundice^a^ | Presence of clinical jaundice with a parasite count >100,000/μL | Clinical jaundice with >100,000 parasites/μL | same |
| Bleeding | Clinical history or examination, and discharge diagnoses. | Significant bleeding | Significant bleeding associated with malaria infection |
| Pulmonary edema | Clinical examination at admission with pulse oximetry when available^b^ | O2 saturation <92% on room air OR respiratory rate >30/min^c^ in the presence of abnormal pulmonary auscultation | same |
| Hypoglycemia | Capillary blood glucose measurement at admission | Capillary blood glucose <2.2mmol/L (<40mg/dL) | same |
| Metabolic acidosis | Not evaluable^d^ | Not evaluable^d^ | Not evaluable^d^ |
| Anemia^a^ | Capillary hematocrit on admission, parasitemia criterion was not applied for *P. vivax* cases | Heemoglobin ≤5g/dL or hematocrit ≤15% in children <12years (<7g/dL and <20% respectively in adults) with >10,000 parasites/μL for *P. falciparum* (not applied for *P. vivax*) | same |
| Hyperparasitemia | Malaria smear parasite count on admission | Hyperparasitemia>10% | same |
| Renal impairment | Discharge diagnoses^d^ | Any diagnosis of renal failure | Any diagnosis of renal failure |

^a^ As per the 2015 WHO malaria guidelines (25) parasitemia thresholds were not applied for *P. vivax*.

^b^ Pulse oximetry was not routinely available until 2012. Severe malaria criteria for pulmonary oedema were adjusted so that oxygen saturation was not a requirement for diagnosing pulmonary edema.

^c^ Increased respiratory rate thresholds were >60/min for infants <2months old, >50/min for infants >2months to <1year old, >40/min for children ≥1year to <5years old, and >30/min if ≥5years old.

^d^ Laboratory tests for this criterion were not performed routinely.

**Supplementary Table 2.**

Hospitalized malaria cases classified as severe by the WHO (2014) research severe malaria criteria (26)

|  | ***Plasmodium vivax*** | | | ***Plasmodium falciparum*** | | |
| --- | --- | --- | --- | --- | --- | --- |
| **Patient characteristics** | **Severe malaria research criteria**  **not met** | **Severe malaria research criteria**  **met** | **p value** | **Severe malaria research criteria**  **not met** | **Severe malaria research criteria**  **met** | **p value** |
|  | N=215,  n (%) | N=63,  n (%) |  | N=3,381,  n (%) | N=1,463,  n (%) |  |
| Infectious diagnosis present | 50 (23) | 20 (32) | 0.17 | 139 (4) | 108 (7) | <0.001 |
| Chronic disease present | 4 (2) | 3 (5) | 0.20 | 8 (0.2) | 10 (0.7) | 0.02 |
| Postpartum females | 11 (8) | 6 (14) | 0.37 | 5 (0.4) | 3 (0.5) | 0.72 |
| Prostration | NA | NA | NA | NA | NA | NA |
| Convulsions | NA | NA | NA | NA | NA | NA |
| Age <1 year | 48 (22) | 8 (13) | 0.63 | 55 (2) | 35 (2) | <0.001 |
| Age 1-5 years | 49 (23) | 15 (24) |  | 715 (21) | 441 (30) |  |
| Age 6-10 years | 14 (7) | 6 (10) | 0.22 | 616 (18) | 272 (19) | <0.001 |
| Age 11-15 years | 8 (4) | 2 (3) |  | 475 (14) | 138 (9) |  |
| Age >15 years | 96 (45) | 32 (51) |  | 1,520 (45) | 577 (39) |  |
| Parasitemia /µL,  Geometric mean (95% CI) | 2556  (32-46,472) | 1556  (32-31,149) | 0.13 | 145,667  (3,520-425,030) | 203,863  (2,556-977,922) | <0.001 |
| Duration of hospital stay, median (IQR, range) days | 3 (2-4, 1-32) | 2 (2-4, 1-26) | 0.9 | 4 (3-6, 1-34) | 5 (4-6, 1-31) | <0.001 |
| Intravenous artesunate | 34 (16) | 10 (16) | 0.99 | 670 (20) | 662 (45) | <0.001 |
| Blood transfusion | 26 (12) | 19 (30) | 0.001 | 324 (10) | 316 (22) | <0.001 |

Data are presented at number (%) unless otherwise indicated.

^a^ The research definition of severe malaria (for both species) does not include prostration or convulsions. See Table 2 for cases that met or did not meet the WHO severe malaria criteria.

^b^ Univariable ordered logistic regression analysis was used to determine relationship between age groups and whether WHO criteria were met.

**Supplementary Table 3.**

Hospitalized malaria cases classified severe by the WHO (2014) research severe malaria criteria (26)

|  | ***P. vivax*** | | | ***P. falciparum*** | | |
| --- | --- | --- | --- | --- | --- | --- |
| **Clinical Characteristics^a^** | **Age ≤15 years** | **Age >15 years** | **Total** | **Age ≤15 years** | **Age >15 years** | **Total** |
|  | **N=31**  **n (%)** | **N=32**  **n (%)** | **N=63**  **n (%)** | **N=886**  **n (%)** | **N=577,**  **n (%)** | **N=1,463,**  **n (%)** |
| Impaired consciousness | 7 (23) | 2 (6) | 9 (14) | 66 (7) | 117 (20) | 183 (13) |
| Prostrate | NA | NA | NA | NA | NA | NA |
| Convulsions | NA | NA | NA | NA | NA | NA |
| Shock | 0 | 4 (13) | 4 (12) | 6 (4) | 28 (5) | 34 (5) |
| Jaundice^b^ | 0 | 0 | 0 | 29 (3) | 82 (15) | 111 (8) |
| Significant bleeding | 0 | 0 | 0 | 0 | 0 | 0 |
| Pulmonary edema | 14 (45) | 13 (41) | 27 (43) | 123 (14) | 44 (8) | 167 (11) |
| Hypoglycemia | 2 (17) | 2 (13) | 4 (14) | 41 (6) | 15 (4) | 56 (5) |
| Metabolic acidosis^c^ | NA | NA | NA | NA | NA | NA |
| Severe anemia^b,d^ | 6 (19) | 11 (34) | 18 (29) | 103 (12) | 64 (11) | 167 (11) |
| Renal impairment^c^ | 0 | 0 | 0 | 1 (0.1) | 7 (1) | 8 (0.6) |
| Hyperparasitemia | NA | NA | NA | 607 (69) | 277 (48) | 884 (60) |
|  |  |  |  |  |  |  |

Data are presented at number (%) unless otherwise indicated.

^a^ 5 cases with *P. vivax* and 263 cases with *P. falciparum* malaria met at least two research severe criteria.

^b^ For *P. vivax* parasite density thresholds were not used in the severity definitions of jaundice or anemia.

^c^ Laboratory testing was not available. For renal impairment the discharge diagnosis was used as a proxy.

^d^ The age threshold used for severe anemia is 12 years old. Severe anemia is defined as hemoglobin ≤5g/dL or hematocrit ≤15% in children <12 years (<7g/dL and <20% respectively in adults)
