# Supplementary material for: Severe Falciparum and Vivax Malaria on the Thailand-Myanmar Border: A Review of 1503 Cases
Source: Clin Infect Dis. 2023 May 5;77(5):721–8. doi: 10.1093/cid/ciad262 (PMC10495127; doi:10.1093/cid/ciad262)
Supplement: Supplementary file 1 [file ciad262_supplementary_data.doc]

Supplementary Table 1.
WHO 2015 severe malaria criteria

**Severe *P. falciparum* malaria criterion**	**How the clinical record was assessed against the WHO criteria**	**Analysis using the WHO (2015) broad definition for severe *P. falciparum* malaria (25)**	**Analysis using the WHO (2014) research definition for severe *P. falciparum* malaria (26)**
Impaired consciousness	GCS and BCS assessment at admission	Glasgow coma score (GCS) <11 in adults or Blantyre coma score <3 in children	same
Prostration	Clinical examination at admission	Unable to walk or sit without assistance	removed
Convulsions	Number of convulsions in the clinical history, and during admission and hospitalization	>2 convulsions in 24 hours	removed
Shock	Systolic blood pressure (SBP) measurement at admission	SBP <80mmHg in adults or <70mmHg in children.	same
Jaundice[a]	Presence of clinical jaundice with a parasite count >100,000/µL	Clinical jaundice with >100,000 parasites/µL	same
Bleeding	Clinical history or examination, and discharge diagnoses.	Significant bleeding	Significant bleeding associated with malaria infection
Pulmonary edema	Clinical examination at admission with pulse oximetry when available[b]	O2 saturation <92% on room air OR respiratory rate >30/min[c] in the presence of abnormal pulmonary auscultation	same
Hypoglycemia	Capillary blood glucose measurement at admission	Capillary blood glucose <2.2mmol/L (<40mg/dL)	same
Metabolic acidosis	Not evaluable[d]	Not evaluable[d]	Not evaluable[d]
Anemia[a]	Capillary hematocrit on admission, parasitemia criterion was not applied for *P. vivax* cases	Hematocrit ≤15% in children <12years (<7g/dL and <20% respectively in adults) with >10,000 parasites/µL for *P. falciparum* (not applied for *P. vivax*)	same
Hyperparasitaemia	Malaria smear parasite count on admission	Hyperparasitemia>10%	same
Renal impairment	Discharge diagnoses[d]	Any diagnosis of renal failure	Any diagnosis of renal failure

[a] As per the 2015 WHO malaria guidelines (25) parasitemia thresholds were not applied for *P. vivax*.
[b] Pulse oximetry was not routinely available until 2012. Severe malaria criteria for pulmonary oedema were adjusted so that oxygen saturation was not a requirement for diagnosing pulmonary edema.
[c] Increased respiratory rate thresholds were >60/min for infants <2months old, >50/min for infants >2months to <1year old, >40/min for children ≥1year to <5years old, and >30/min if ≥5years old.
[d] Laboratory tests for this criterion were not performed routinely.

Supplementary Table 2.
Hospitalized malaria cases classified as severe by the WHO (2014) research severe malaria criteria (26)

Patient characteristics	*Plasmodium vivax*			*Plasmodium falciparum*		
	**Severe malaria research criteria not met**	**Severe malaria research criteria met**	**p value**	**Severe malaria research criteria not met**	**Severe malaria research criteria met**	**p value**
	N=215, n (%)	N=63, n (%)		N=3,381, n (%)	N=1,463, n (%)	
Infectious diagnosis present	50 (23)	20 (32)	0.17	139 (4)	108 (7)	<0.001
Chronic disease present	4 (2)	3 (5)	0.20	8 (0.2)	10 (0.7)	0.02
Postpartum females	11 (8)	6 (14)	0.37	5 (0.4)	3 (0.5)	0.72
Prostration	NA	NA	NA	NA	NA	NA
Convulsions	NA	NA	NA	NA	NA	NA
Age <1 year	48 (22)	8 (13)	0.63	55 (2)	35 (2)	<0.001
Age 1-5 years	49 (23)	15 (24)		715 (21)	441 (30)	
Age 6-10 years	14 (7)	6 (10)	0.22	616 (18)	272 (19)	<0.001
Age 11-15 years	8 (4)	2 (3)		475 (14)	138 (9)	
Age >15 years	96 (45)	32 (51)		1,520 (45)	577 (39)	
Parasitemia /µL, Geometric mean (95% CI)	2556 (32-46,472)	1556 (32-31,149)	0.13	145,667 (3,520-425,030)	203,863 (2,556-977,922)	<0.001
Duration of hospital stay, median (IQR, range) days	3 (2-4, 1-32)	2 (2-4, 1-26)	0.9	4 (3-6, 1-34)	5 (4-6, 1-31)	<0.001
Intravenous artesunate	34 (16)	10 (16)	0.99	670 (20)	662 (45)	<0.001
Blood transfusion	26 (12)	19 (30)	0.001	324 (10)	316 (22)	<0.001

Data are presented at number (%) unless otherwise indicated.
[a] The research definition of severe malaria (for both species) does not include prostration or convulsions. See Table 2 for cases that met or did not meet the WHO severe malaria criteria.
[b] Univariable ordered logistic regression analysis was used to determine relationship between age groups and whether WHO criteria were met.

Supplementary Table 3.
Hospitalized malaria cases classified severe by the WHO (2014) research severe malaria criteria (26)

Clinical Characteristics[a]	*P. vivax*			*P. falciparum*		
	**Age ≤15 years**	**Age >15 years**	**Total**	**Age ≤15 years**	**Age >15 years**	**Total**
	**N=31 n (%)**	**N=32 n (%)**	**N=63 n (%)**	**N=886 n (%)**	**N=577, n (%)**	**N=1,463, n (%)**
Impaired consciousness	7 (23)	2 (6)	9 (14)	66 (7)	117 (20)	183 (13)
Prostrate	NA	NA	NA	NA	NA	NA
Convulsions	NA	NA	NA	NA	NA	NA
Shock	0	4 (13)	4 (12)	6 (4)	28 (5)	34 (5)
Jaundice[b]	0	0	0	29 (3)	82 (15)	111 (8)
Significant bleeding	0	0	0	0	0	0
Pulmonary edema	14 (45)	13 (41)	27 (43)	123 (14)	44 (8)	167 (11)
Hypoglycemia	2 (17)	2 (13)	4 (14)	41 (6)	15 (4)	56 (5)
Metabolic acidosis[c]	NA	NA	NA	NA	NA	NA
Severe anemia[b,d]	6/29 (21)	12/34 (35)	18/63 (29)	95/775 (12)	72/688 (10)	167/1,463 (11)
Renal impairment[c]	0	0	0	1 (0.1)	7 (1)	8 (0.6)
Hyperparasitemia	NA	NA	NA	607 (69)	277 (48)	884 (60)

Data are presented at number (%) unless otherwise indicated.
[a] 5 cases with *P. vivax* and 263 cases with *P. falciparum* malaria met at least two research severe criteria.
[b] For *P. vivax* parasite density thresholds were not used in the severity definitions of jaundice or anemia.
[c] Laboratory testing was not available. For renal impairment the discharge diagnosis was used as a proxy.
[d] The age threshold used for severe anemia is 12 years old in the WHO malaria guidelines. The denominator is the number of patients who meet the research definition of the severe malaria criteria and are below or above 12 years.
